# Association between drinking water type and kidney stone risk in U.S. adults: a cross-sectional analysis of NHANES 2009–2016 data

**DOI:** 10.1097/JS9.0000000000003248

**Published:** 2025-08-21

**Authors:** Jiajie Qin, Jiyuan Li, Hedong Zhang, Hao Wen, Ye Xu, Qiulin Luo, Jiawei Peng, Tengfang Li, Xiangqi Zhang, Rongzheng Nie, Longkai Peng, Helong Dai

**Affiliations:** a Department of Kidney Transplantation, Center of Organ Transplantation, The Second Xiangya Hospital of Central South University, Changsha, Hunan Province, China; b Department of Software Engineering, School of Software, Beihang University, Beijing, China; c Medical College of Guangxi University, Nanning, Guangxi Province, China; d Department of Immunology, School of Basic Medical Science, Central South University, Changsha, Hunan Province, China

**Keywords:** bottled water, drinking water, fluid intake, kidney stone, NHANES, tap water

## Abstract

**Purpose:**

While fluid intake and trace elements are known to influence kidney stone risk, the impact of drinking water type remains unclear. This study aimed to systematically evaluate the association between water source and kidney stone prevalence.

**Methods:**

This analysis included 10 246 adults (≥20 years) from the National Health and Nutrition Examination Survey database (2009–2016). Kidney stone history was self-reported, and drinking water sources were assessed through 24-hour dietary recalls.

We employed multivariable-adjusted logistic regression models, adjusting for potential confounders including age, sex, race, body mass index (BMI), physical activity, calcium intake, smoking status, alcohol consumption, diabetes, and hypertension, to evaluate the association between drinking water type and kidney stone occurrence. To assess potential nonlinear relationships, restricted cubic spline (RCS) models were utilized. Additionally, cluster analysis was performed to categorize participants into distinct subgroups based on water consumption patterns. Finally, propensity score matching (PSM) and subgroup analyses were conducted to further validate the robustness of the findings.

**Results:**

Logistic regression suggested that higher tap water intake was significantly inversely related with kidney stone prevalence [adjusted odds ratio (OR) = 0.678, 95% confidence interval (CI): 0.491–0.938, *P* for trend = 0.0177], suggesting a 32% lower odds of kidney stones with increased tap water consumption. However, bottled water intake was not significantly associated with kidney stone risk. The RCS model revealed a linear decreasing trend between tap water intake and kidney stone risk (*P* for all <0.0001, *P* for nonlinear = 0.8505). Cluster analysis showed that high tap water intake and low bottled water intake were associated with a lower prevalence of kidney stones. This association persisted in the propensity score-matched models and subgroup analyses after adjustment for bottled water intake.

**Conclusion:**

Our study revealed an inverse association between tap water intake and kidney stone prevalence among U.S. adults. Bottled water intake had no statistically significant effect on stone formation. These results suggest that higher tap water intake may confer a protective effect against kidney stone formation. These findings could guide public health recommendations on hydration habits.

**HIGHLIGHTS**

- This article presents the first large-scale evidence delineating the dose‒response relationship between drinking water type (tap vs. bottled) and nephrolithiasis incidence using big-data analytics.
- Through an integrated analytical approach combining cluster analysis, restricted cubic spline (RCS) modelling, and propensity score matching (PSM), we demonstrated a consistent inverse association between tap water intake and nephrolithiasis risk.
- These findings provide clinically actionable guidance for both reducing recurrence in both stone formers and the general population, underscoring the importance of personalized water selection strategies on the basis of mineral composition.

## Introduction

Kidney stones have emerged as a globally prevalent health concern, with prevalence rates demonstrating a marked increase over recent decades[1]. Over the past decade, prevalence rates remained stable in males (11.9% in 2017–2018) while showing a significant increase in females (rising from 6.5% to 9.4% during the same period)^[2,3]^. The management of kidney stones imposes a dual burden on healthcare systems and economies[4]. The pathogenesis involves multifactorial interactions, including metabolic disorders and dietary patterns (e.g., high sodium and animal protein intake)^[5–8]^. Notably, a bidirectional association exists between metabolic diseases (obesity, diabetes, hypertension) and kidney stones: these conditions not only serve as independent risk factors for stone formation but also predispose kidney stone patients to an elevated risk of developing chronic kidney disease (CKD)^[9,10]^.

A well-established relationship exists between fluid intake and kidney stone risk. Substantial evidence indicates that adequate hydration reduces both the primary prevalence and recurrence rates of kidney stones by promoting urinary dilution and decreasing the supersaturation of lithogenic solutes^[11–14]^. While public attention to drinking water sources and their health implications has grown substantially, a significant proportion of adults fail to meet recommended daily fluid intake levels, posing both individual and public health challenges^[15–17]^.

Recent studies indicate that certain mineral components in water, especially calcium and magnesium, may have either inhibitory or promotive effects on kidney stone formation. Additionally, bicarbonate-rich water appears to be linked to lower rates of uric acid stones^[18,19]^. Research has explored the potential roles of water components in modulating stone formation risk and preventing kidney stones^[20–22]^. However, whether different types of drinking water exert distinct effects on kidney stone development remains to be elucidated. Utilizing the nationally representative National Health and Nutrition Examination Survey (NHANES) data, our study provides robust epidemiological evidence comparing the effects of tap water versus bottled water consumption on kidney stone formation risk. These findings offer direct evidence to inform public health recommendations for stone prevention.

This cross-sectional has been reported in line with the STROCSS criteria[23].

## Methods

### Study population

**Figure 1.**
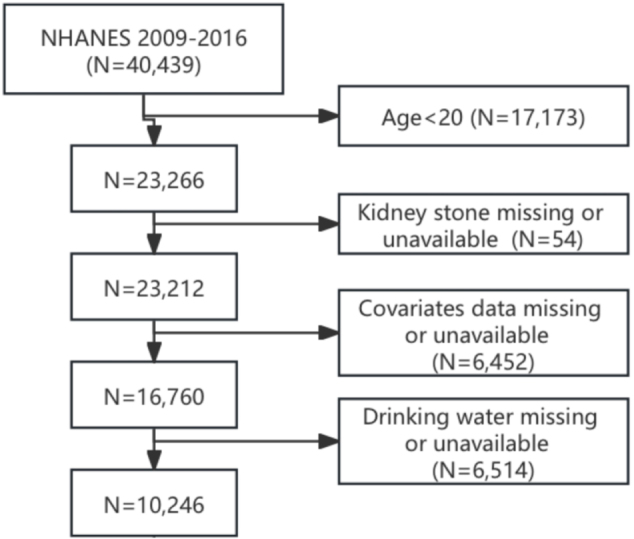
Flowchart of participant screening.

The study sample was derived from the NHANES, a nationally representative cross-sectional program conducted by the Centers for Disease Control and Prevention (CDC). The NHANES is an ongoing biennial, nationally representative cross-sectional survey that comprehensively assesses the health and nutritional status of the U.S. population through standardized interviews, physical examinations, and laboratory tests. For this analysis, we included participants from four consecutive NHANES cycles (2009–2016), with a final cohort of 10 246 eligible adults after applying the following exclusion criteria (Fig. 1): (1) age <20 years; (2) missing kidney stone history data; (3) incomplete covariate information; and (4) unavailable drinking water intake data. The NHANES protocol was approved by the National Center for Health Statistics (NCHS) Research Ethics Review Board. All participants provided written informed consent. As this secondary analysis used de-identified public data, it was exempt from additional institutional review board approval[24].

### Drinking water source

Drinking water source served as the primary exposure variable in our analysis. Dietary intake data were collected through two 24-hour recall interviews: the first was conducted in person at mobile examination centers (MECs), followed by a telephone-administered second recall 3–10 days later. We calculated the mean daily water intake values by averaging the reported consumption from both interviews. The NHANES classifies drinking water sources into two categories: tap water (including filtered tap water and water from a drinking fountain) and bottled water.

### Kidney stone history

The presence of kidney stones served as the primary outcome variable in our study. Trained interviewers ascertained kidney stone history using the computer-assisted personal interview (CAPI) system during MEC visits. Participants who responded affirmatively to the standardized question “Have you ever had kidney stones?” were classified as having a positive kidney stone history.

### Definitions of variables

We adjusted for potential confounding factors through multivariable modelling, incorporating the following covariates: age (20–40 years, 40–60 years, >60 years); sex (male/female); race (Mexican American, other Hispanic, non-Hispanic White, non-Hispanic Black, other race); BMI (≤25 kg/m^2^, 25–30 kg/m^2^, > 30 kg/m^2^); physical activity (vigorous, moderate, or less than moderate exercise); calcium intake; smoking status (no/yes); alcohol consumption (no/yes); diabetes (no/yes); and hypertension (no/yes)^[25,26]^.

### Statistical analysis

This study employed weighted analyses to account for the complex multistage probability sampling design of the NHANES, ensuring national representativeness. Continuous variables are presented as the means ± standard deviations (SD), while categorical variables are expressed as percentages (%). Group comparisons were performed using analysis of variance (ANOVA) for continuous variables and chi-square tests for categorical variables. Drinking water consumption was categorized into quintiles (Q1–Q5) for both tap water and bottled water, with Q1 serving as the reference group. We conducted weighted multivariable logistic regression analyses using two models: a crude model without adjustments and a fully adjusted model incorporating age, sex, race/ethnicity, BMI, physical activity, calcium intake, smoking status, alcohol consumption, diabetes status, and hypertension. The results are reported as odds ratios (ORs) with 95% confidence intervals (CIs), with trend tests performed across water intake quintiles.

To examine potential nonlinear relationships, we employed restricted cubic splines (RCSs) with three knots. Cluster analysis using the *k*-means algorithm was performed to identify distinct patterns of water consumption behavior. For robustness assessment, we conducted propensity score matching (PSM) with 1:1 nearest-neighbor matching and a caliper width of 0.05 to balance potential confounders, followed by subgroup analyses to evaluate the consistency of the observed associations.

All the statistical analyses were performed using R software (version 4.3.3), with two-tailed *P* values <0.05 considered statistically significant.

## Results

### Baseline characteristics

The study included 10 246 participants with complete data (Table 1), of whom 980 (9.6%) had kidney stones. When participants were grouped by kidney stone status, most baseline characteristics were significantly different. Compared with the nonstone group, the stone-forming group was older (51% aged 60–80 years); was more likely to be male (56%); had a higher proportion of non-Hispanic whites (61%); had a higher BMI (46% with BMI > 30 kg/m^2^); had lower physical activity levels (62% with low activity); and had higher rates of smoking (48%), diabetes (3.2%), and hypertension (53%). More importantly, kidney stone patients had lower tap water intake (871 g) and higher bottled water consumption (954 g).

**Table 1:** Baseline characteristics of participants between 2009 and 2016 (*n* = 10 246)

Characteristic	Kidney stone			
	Total	No	Yes	*P*-value
*N*	10 246	9266 (90%)	980 (9.6%)	
Age, *n* (%)				<0.001
20–40 years	3243 (32%)	3078 (33%)	165 (17%)	
40–60 years	3307 (32%)	2993 (32%)	314 (32%)	
60–80 years	3696 (36%)	3195 (34%)	501 (51%)	
Gender, *n* (%)				<0.001
Male	5012 (49%)	4461 (48%)	551 (56%)	
Female	5234 (51%)	4805 (52%)	429 (44%)	
Race/ethnicity, *n* (%)				<0.001
Mexican American	1093 (11%)	988 (11%)	105 (11%)	
Other Hispanic	925 (9.0%)	815 (8.8%)	110 (11%)	
Non-Hispanic White	5128 (50%)	4535 (49%)	593 (61%)	
Non-Hispanic Black	1775 (17%)	1672 (18%)	103 (11%)	
Other race/Multiracial	1325 (13%)	1256 (14%)	69 (7.0%)	
BMI (kg/m^2^)				<0.001
<25	3070 (30%)	2879 (31%)	191 (19%)	
25–30	3363 (33%)	3027 (33%)	336 (34%)	
>30	3813 (37%)	3360 (36%)	453 (46%)	
Physical Activity, *n* (%)				<0.001
Vigorous	1697 (17%)	1585 (17%)	112 (11%)	
Moderate	2935 (29%)	2673 (29%)	262 (27%)	
Low	5614 (55%)	5008 (54%)	606 (62%)	
Smoking status, *n* (%)				<0.001
Never	5940 (58%)	5429 (59%)	511 (52%)	
Ever	4306 (42%)	3837 (41%)	469 (48%)	
Alcohol status, *n* (%)				0.7
No	2743 (27%)	2476 (27%)	267 (27%)	
Yes	7503 (73%)	6790 (73%)	713 (73%)	
Diabetes, *n* (%)				0.12
No	9997 (98%)	9048 (98%)	949 (97%)	
Yes	249 (2.4%)	218 (2.4%)	31 (3.2%)	
Hypertension, *n* (%)				<0.001
No	6419 (63%)	5955 (64%)	464 (47%)	
Yes	3827 (37%)	3311 (36%)	516 (53%)	
Calcium intake (mg)	936 ± 490	940 ± 493	903 ± 457	0.045
Tap water intake (g)	956 ± 931	965 ± 936	871 ± 878	<0.001
Bottled water intake (g)	927 ± 827	924 ± 820	954 ± 894	0.8

### Association between drinking water sources and kidney stone risk

The study categorized drinking water intake into quintiles, with tap water consumption divided as Q1 (≤262.5 g/day), Q2 (262.5–592.5 g/day), Q3 (592.5–982.5 g/day), Q4 (982.5–1672.5 g/day), and Q5 (>1672.5 g/day), and bottled water intake classified as Q1 (≤253.5 g/day), Q2 (253.5–507.0 g/day), Q3 (507.0–887.3 g/day), Q4 (887.3–1494.0 g/day), and Q5 (>1494.0 g/day).

**Figure 2.**
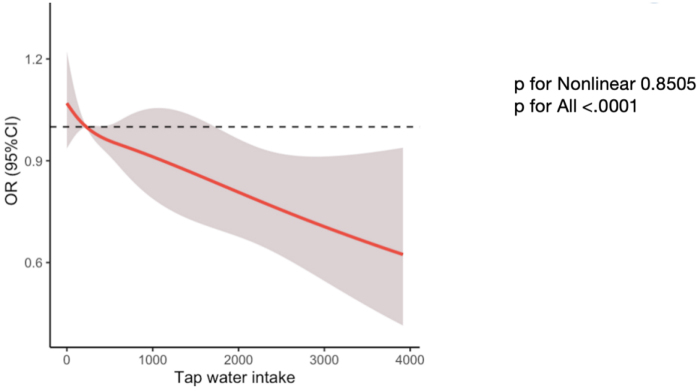
RCS curves describing the dose‒response relationship between tap water intake and kidney stone. The following covariates were adjusted for: age, sex, race, BMI, physical activity, calcium intake, smoking status, diabetes mellitus, hypertension, and bottled water intake. BMI, body mass index; RCS, restricted cubic spline.

Univariate logistic regression analysis revealed a significant inverse association between tap water consumption and kidney stone risk (crude OR = 0.983, *P* = 0.0017) (Table 2), demonstrating a clear dose‒response relationship with decreasing risk at higher intake levels (*P* for trend <0.001). This protective association remained statistically significant after multivariate adjustment for age, sex, race, BMI, physical activity, calcium intake, smoking status, diabetes, hypertension, and bottled water intake (adjusted OR = 0.986, 95% CI = 0.976–0.997, *P* = 0.0136). RCS analysis confirmed a linear relationship between tap water intake and kidney stone risk (*P* for all <0.0001, *P* for nonlinear = 0.8505, Fig. 2). Supplemental Digital Content Figure 1, available at: http://links.lww.com/JS9/E904 further supported a decreasing trend in kidney stone prevalence with increasing tap water intake. Notably, bottled water intake was not significantly associated with kidney stone risk according to either the unadjusted or adjusted model (Table 3).

**Table 2:** Univariate and multivariate logistic regression analyses by the association between kidney stone and tap water intake, weighted

Characteristic	Kidney stone infection crude OR (95% CI)	*P* for trend	Kidney stone infection adjusted OR (95% CI)	*P* for trend
Continuous tap water intake per 100 g increase	0.983 (0.980, 0.996)	0.0017	0.986 (0.976, 0.997)	0.0136
Categorical tap water intake		<0.001		0.0177
Q1	Ref		Ref	
Q2	0.794 (0.604, 1.044)		0.800 (0.600, 1.064)	
Q3	0.689 (0.527, 0.900)		0.714 (0.541, 0.941)	
Q4	0.736 (0.555, 0.975)		0.822 (0.632, 1.069)	
Q5	0.603 (0.452, 0.805)		0.678 (0.491, 0.938)

**Table 3:** Univariate and multivariate logistic regression analyses by the association between kidney stone and bottled water intake, weighted

Characteristic	Kidney stone infection crude OR (95% CI)	*P* for trend	Kidney stone infection adjusted OR (95% CI)	*P* for trend
Continuous bottled water intake per 100 g increase	1.005 (0.992, 1.018)	0.463	1.009 (0.995, 1.023)	0.222
Categorical bottled water intake		0.327		0.188
Q1	Ref		Ref	
Q2	0.935 (0.681, 1.283)		0.948 (0.670, 1.341)	
Q3	1.077 (0.798, 1.454)		1.104 (0.796, 1.531)	
Q4	1.152 (0.847, 1.568)		1.196 (0.859, 1.666)	
Q5	1.105 (0.767, 1.591)		1.194 (0.808, 1.765)	

### Cluster analysis-derived drinking water patterns

**Figure 3.**
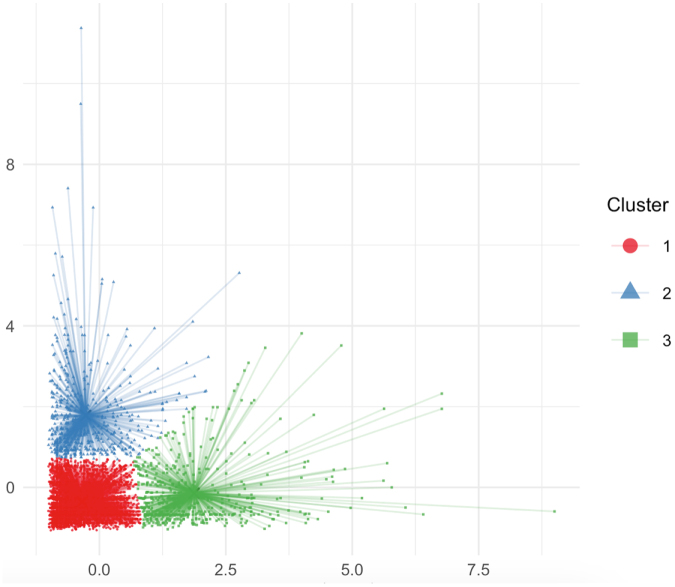
Visualization of *k*-mean clustering. Cluster 1 (red) double-low group; Cluster 2 (blue) high bottled water group; Cluster 3 (green) high tap water group; we defined Cluster 1 (red) with low tap water intake and low bottled water intake as the double-low group; Cluster 2 (blue) with low tap water intake and high bottled water intake as the high bottled water group; and Cluster 3 (green) with high tap water intake and low bottled water intake as the high tap water group.

Through *k*-means clustering analysis (*k* = 3), the participants were classified into groups representing three distinct drinking water patterns (Fig. 3). Cluster 1 was characterized as the “Double-Low Group.” Cluster 2 represented the “High Bottled Water Group,” whereas Cluster 3 was identified as the “High Tap Water Group.” The centroid values for each cluster are detailed in Supplemental Digital Content Table 1, available at: http://links.lww.com/JS9/E904.

### Kidney stone risk associated with drinking water patterns

**Figure 4.**
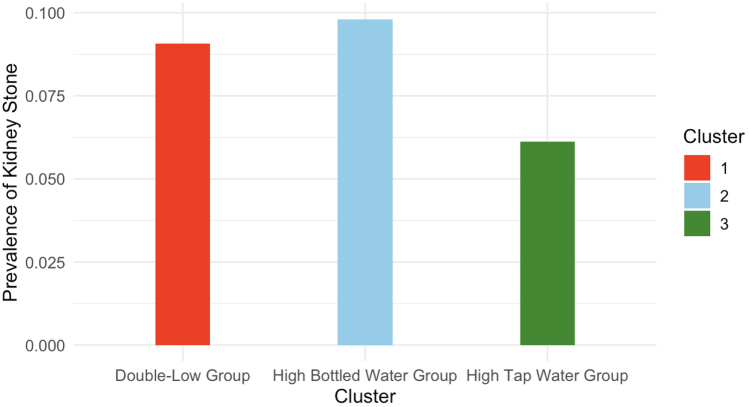
Prevalence of the three clusters.

As demonstrated in Figure 4, the cluster with high tap water intake presented a significantly lower prevalence of kidney stones than the other groups did (Prevalence of kidney stones = 0.0611), Interestingly, the group with high bottled water intake presented a greater prevalence of kidney stones than the other groups did (Prevalence of kidney stones = 0.0981). In the unadjusted logistic regression model (Table 4) with Cluster 1 as a reference, Cluster 2 demonstrated a decreased risk of kidney stones (crude OR = 0.652, 95% CI = 0.442–0.963, *P* = 0.0315). These results suggest that increased tap water intake may confer protective effects against kidney stone formation. On the basis of cumulative evidence, we recommend prioritizing tap water over bottled water for daily hydration needs in kidney stone prevention strategies.

**Table 4:** Univariate and multivariate logistic regression analyses by the association between the clusters

Characteristic	Kidney stone infection crude OR (95% CI)	*P*-value	Kidney stone infection adjusted OR (95% CI)	*P*-value
Cluster 1	Ref		Ref	
Cluster 2	1.089 (0.790, 1.502)	0.6009	1.237 (0.886, 1.725)	0.2113
Cluster 3	0.652 (0.442, 0.963)	0.0315	0.764 (0.513, 1.137)	0.1842

### Sensitivity analyses

#### Comparison after PSM

To increase the robustness of our findings and better control for potential confounding effects of bottled water intake, we performed PSM on the basis of bottled water intake levels. Both univariate and multivariate logistic regression analyses conducted in the matched cohort replicated our primary results (Table 5). The persistent inverse association between tap water intake and kidney stones prevalence (adjusted OR = 0.563, 95% CI: 0.367–0.864, *P* for trend = 0.0022) reinforces the protective effect observed in our initial analyses.

**Table 5:** Univariate and multivariate logistic regression analyses by the association between kidney stone and tap water intake after PSM, weighted

Characteristic	Kidney stone infection crude OR (95% CI)	*P* for trend	Kidney stone infection adjusted OR (95% CI)	*P* for trend
Continuous tap water intake per 100 g increase	0.983 (0.971, 0.996)	0.01	0.987 (0.974, 0.999)	0.0423
Categorical tap water intake		<0.001		0.0022
Q1	Ref		Ref	
Q2	0.903 (0.614, 1.329)		0.891 (0.580, 1.370)	
Q3	0.693 (0.506, 0.949)		0.695 (0.502, 0.961)	
Q4	0.679 (0.472, 0.976)		0.732 (0.500, 1.070)	
Q5	0.538 (0.366, 0.791)		0.563 (0.360, 0.864)	

#### Subgroup analysis

**Figure 5.**
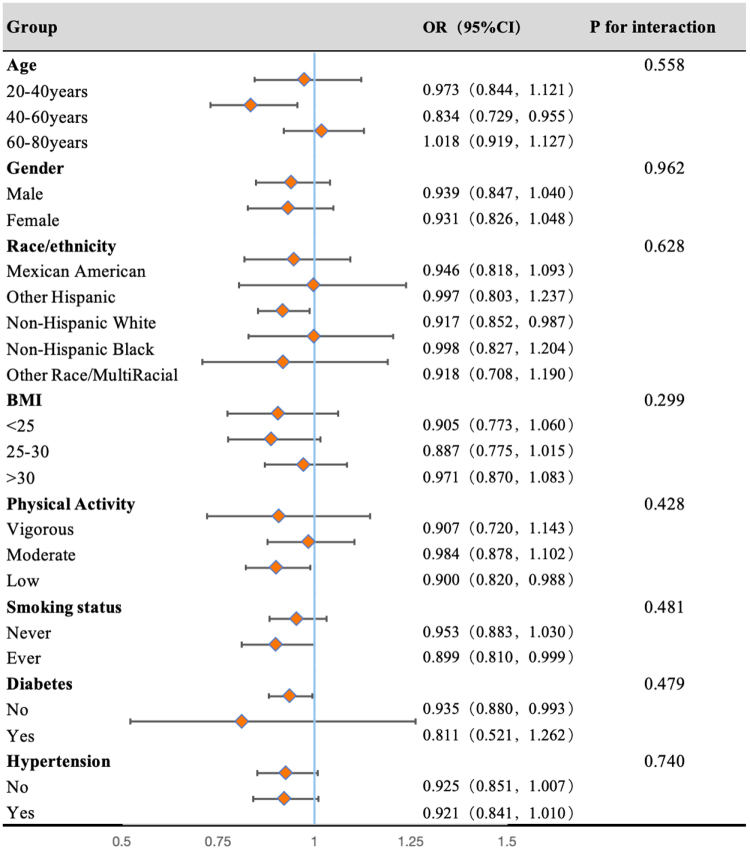
Forest plots for subgroup analysis. Subgroup analysis was stratified by age, sex, race, BMI, physical activity, smoking status, diabetes, and hypertension. BMI, body mass index.

We conducted comprehensive subgroup analyses with forest plot visualization (Fig. 5) to examine potential effect modifications between stone risk and categorical variables. The results demonstrated a consistent inverse association between tap water intake and kidney stones prevalence across the majority of subgroups stratified by demographic characteristics, lifestyle factors, and comorbid conditions (all *P* for interaction >0.05).

## Discussion

This cross-sectional study investigated the associations between drinking water sources and kidney stones prevalence among 10 246 U.S. adults and revealed an overall stone prevalence of 9.6%. Multivariate logistic analyses revealed a significant independent inverse association between tap water intake and kidney stone risk, with strengthened protective effects observed across increasing intake quintiles (*P* for trend <0.001). Restricted cubic spline (RCS) analyses confirmed a dose‒dependent negative correlation. Cluster analysis further identified three distinct water intake patterns, with the “High Tap Water Group” showing the lowest stone risk. These findings remained robust in propensity score-matched analyses (caliper = 0.05) and subgroup analysis, whereas bottled water intake and kidney stone risk were not significantly associated. The consistent protective relationship suggests that choosing tap water for daily hydration may be a simple yet effective strategy for the primary prevention of kidney stones in the general population.

The protective effect of high fluid intake against kidney stones has been well documented in previous studies^[27–30]^, with consistent evidence indicating that increased water intake is an effective preventive measure. Emerging research has further elucidated the dual role of drinking water components in both promoting and inhibiting the risk of stone formation^[21,30–35]^. Our findings may be explained by the differential mineral composition across water types: treated tap water typically contains optimal concentrations of calcium and magnesium, and calcium binds with dietary oxalate in the intestine to form insoluble calcium oxalate, thereby reducing oxalate absorption and subsequent urinary excretion^[36–38]^. In contrast, commercially available bottled water has substantial variability in mineral content, often lacking these protective components or containing imbalanced ratios that may negate potential benefits^[18,39–41]^. Furthermore, behavioural differences between healthy individuals and stone formers may contribute to this association, as patients with kidney stones tend to demonstrate lower thirst perceptions^[42–44]^ and may preferentially consume bottled water for convenience, potentially compounding their risk through suboptimal hydration practices.

Our findings suggest that tap water may represent a healthier choice for kidney stone prevention, although several limitations warrant consideration. First, the generalizability of this conclusion may vary across regions due to differences in municipal water supplies and the prevalence of distinct bottled water brands with varying mineral compositions. Notably, our analysis lacked direct measurements of tap/bottled water mineral content. Second, as a cross-sectional study, our design cannot establish causality, highlighting the need for longitudinal studies to evaluate the long-term clinical outcomes associated with different drinking water sources. Third, residual confounding persists despite multivariable adjustment – key factors could partially explain the observed associations. While current evidence indicates that tap water may be superior to bottled water for kidney stones prevention, definitive confirmation of this association requires well-designed intervention studies with standardized water quality monitoring profiling to assess crystallization risk. Notably, breakthroughs in novel drug delivery systems (NDDS) provide complementary therapeutic approaches for stone management. These advanced delivery platforms have shown promise in improving the bioavailability, targeted delivery, and therapeutic efficacy of litholytic or anti-inflammatory agents, potentially reducing recurrence rates. The integration of these technological advancements with our findings may offer a more comprehensive strategy for stone prevention^[45–48]^.

Our study leverages the nationally representative NHANES database, which employs standardized protocols and rigorous quality control measures conducted by trained professionals to ensure data reliability. The large sample size (*n* = 10 246) and appropriate sampling weights increase the statistical power and generalizability of our findings. These methodological advantages support the robustness of our primary conclusion regarding the associations between drinking water sources and kidney stones risk. From a public health perspective, these evidence-based findings provide meaningful guidance for hydration recommendations in kidney stone prevention.

## Conclusion

Our study revealed a significant inverse association between tap water intake and the prevalence of kidney stones among U.S. adults, whereas no such protective effect was observed with bottled water intake. These findings suggest that increased tap water intake may serve as a modifiable protective factor against kidney stones. From a public health perspective, prioritizing tap water over bottled water for daily hydration may serve as a simple and practical strategy to prevent kidney stones in the general population.

## Data Availability

The data supporting the findings of this study were obtained from the National Health and Nutrition Examination Survey (NHANES) and are publicly accessible at: https://www.cdc.gov/nchs/nhanes/index.htm.

## Research registration unique identifying number (UIN)

In view of the fact that our research focuses on the analysis of public databases, there is no Unique Identifying Number (UIN) available for submission.
